# Gender discrepancy of incidence and risk factors of metabolic syndrome among rural Chinese from 2012–2013 to 2015–2017

**DOI:** 10.1186/s13098-020-00542-2

**Published:** 2020-06-03

**Authors:** Shasha Yu, Xiaofan Guo, GuangXiao Li, Hongmei Yang, Guozhe Sun, Liqiang Zheng, Yingxian Sun

**Affiliations:** 1 Department of Cardiology, First Hospital of China Medical University, Shenyang, 110001 China; 2 Department of Clinical Epidemiology, Institute of Cardiovascular Diseases, First Hospital of China Medical University, Shenyang, 110001 China; 3 Department of Clinical Epidemiology, Shengjing Hospital of China Medical University, Shenyang, 110004 China

**Keywords:** Incidence, MetS, Metabolic disorders, Gender, Discrepancy, Dietary changes

## Abstract

**Background:**

We reported a relatively high rate of MetS in rural Northeast residents in 2012–2013. Many strategies like health knowledge propagation and lifestyle modification have been taken to help rural residents decrease metabolic disorders. Hence, we held the present follow-up study in order to figure the changes of metabolic parameters and the possible reasons together with the evaluation of MetS incidence and associated risk factors.

**Methods:**

A population-based sample of 8147 rural Northeast Chinese residents aged ≥ 35 years at baseline were followed up from 2012–2013 to 2015–2017. MetS was diagnosed following the unify criteria in 2009 using the Asian specific criteria.

**Results:**

Among residents with MetS at baseline, value of systolic, diastolic blood pressure, total cholesterol, HDL-C decreased while waist circumference increased in both genders in follow-up. Discrepancy of trend in body mass index, LDL-C and estimated GFR existed between male and female. Besides, triglyceride increased, and fast glucose decreased in female only. The alterations of dietary pattern might be accountable for those changes. Among residents without MetS at baseline, the cumulative incidence of newly diagnosed MetS was 24.0% (25.8% for male; 22.3% for female). As the number of metabolic disorders increased at baseline, the incidence of MetS also increased (zero metabolic disorder: 8.3%; one metabolic disorder: 17.1%; two metabolic disorders: 35.4%). In male residents, bad living habits like smoking and drinking were associated with increasing risk of Mets while in female, higher risk of MetS was more likely relevant to dietary pattern.

**Conclusion:**

Metabolic parameters changes during the past years and seem to be associated with alteration of diet pattern. Incidence of MetS still high among rural Northeast Chinese. The risk factors of higher incidence of MetS show gender discrepancy which make the prophylaxis and control of MetS more effective and directive in rural residents.

## Background

MetS is the cluster of metabolic disorders that include elevated blood pressure, abdominal obesity, insulin resistance, dyslipidemia, and is strongly associated with increased risk of many diseases like twofold for cardiovascular disease and 5- fold or more for type 2 diabetes [1, 2]. Besides, in recent year, growing evidence claimed that MetS was relevant to many other kinds of diseases, like breast cancer, hypothyroidism and cerebral microbleeds [3–5]. MetS has already become a huge potential risk factors for public health. Out previous study reported the high prevalence (39.0%) of MetS among rural Northeast Chinese (45.6% for female; 31.4% for male). There are growing propagation about health lifestyle modification and investment of medical source on public health in China. However, the incidence of MetS still increased from 8 to 10.6% in urban areas and 4.9 to 5.3% in rural areas [6]. Previous study reported that during 1988–2010, there is a significantly increase of waist circumference (WC) in general population in USA [7]. While other study in Ahvaz claimed that lipid profile like HDL, triglycerides and Cholesterol decreased from 2009 to 2014 among general subjects over 20 years old [8]. However, there is lack of study focus on the metabolic changes among previous diagnosed MetS. Whether their metabolic parameters improved or worse during the past years. Hence, one aim of the present study is to estimate the changes of metabolic parameters between 2012–2013 and 2015–2017. Through this investigation we can figure out whether the strategies like health knowledge propagation and lifestyle modification works or not. Second, to estimate the cumulative incidence of MetS between 2012–2013 and 2015–2017 and found the possible risk factors for better control.

## Materials and methods

### Study population

The Northeast China Rural Cardiovascular Health Study (NCRCHS) is a community-based prospective cohort study carried out in rural areas of Northeast China. The design and inclusion criteria of the study has been described previously [9, 10]. In brief, a total of 11,956 participants aged ≥ 35 years were recruited from Dawa, Zhangwu and Liaoyang counties in Liaoning province between 2012 and 2013, using a multi-stage, randomly stratified cluster-sampling scheme. The study was approved by the Ethics Committee of China Medical University (Shenyang, China AF-SDP-07-1, 0-01). Detailed information was collected at baseline for each participant. In 2015 and 2017, participants were invited to attend a follow-up study. Of the 11,956 subjects, 1256 participants were not included due to missing contact information and 10,349 participants (86.6%) completed at least one follow-up visit. The median follow-up was 4.66 years. The study was approved by the Ethics Committee of China Medical University (Shenyang, China). Written informed consent was obtained from all participants. In the current analyses, we excluded participants with a history of stroke (n = 387), coronary heart disease (CHD, n = 520) or heart failure at baseline. Complete information on co-variables from the baseline visit was required for inclusion in the current analyses. Finally, data were available for 8147 participants.

### Study variables

At baseline, detailed information on demographic characteristics, dietary and lifestyle factors and medical history were obtained by interview with a standardized questionnaire. Smoking and drinking status were defined as current use. Dietary pattern included were assessed by residents recall the foods that they eat. The questionnaire included questions regarding the average consumption of different food items per week. Vegetable consumption was assessed on the following scale: rarely, < 1000 g, 1000–1500 g, 1500–2000 g, > 2000 g; Meat consumption, including red meat, fish and poultry was assessed on the following scale: rarely, < 250 g, 250–500 g, > 500 g; Bean and bean product consumption was assessed on the following scale: rarely, 2–3times, ≥ 4 times; Greasy food consumption was assessed on the following scale: rarely, 2–3 times, ≥ 4 times; Tea consumption: No, occasionally, 1–2 times, ≥ 3 times; Physical activity contained occupational and leisure-time physical activity. We have detailed descripted it previously [9]. It is divided into three classes, low, moderate and heavy. History of stroke, CHD and heart failure at baseline was defined as self-reported and confirmed by medical records. Weight and height were measured with participants in light weight clothing and without shoes. Waist circumference was measured at the umbilicus using a non-elastic tape. Body mass index (BMI) was computed as weight in kilograms divided by the square of height in meters. Obesity was defined as BMI ≥ 28 kg/m^2^ [11]. Blood pressure was assessed three times with participants seated after at least 5 min of rest using a standardized automatic electronic sphygmomanometer (HEM-907; Omron, Tokyo, Japan). Hypertension was defined as systolic blood pressure (SBP) ≥ 140 mm Hg and/or diastolic blood pressure (DBP) ≥ 90 mm Hg, and/or use of antihypertensive medications [12]. Fasting blood samples were collected in the morning from participants who had fasted at least 12 h. Fasting plasma glucose (FPG), total cholesterol (TC), low-density lipoprotein cholesterol (LDL-C), high-density lipoprotein cholesterol (HDL-C), triglyceride (TG), serum creatinine and other routine blood biochemical indexes were analyzed enzymatically. Estimated glomerular filtration rate (eGFR) was calculated using the Chronic Kidney Disease Epidemiology Collaboration (CKD-EPI) equation [13]. MetS was diagnosed follow the unify criteria from the meeting between several major organizations in 2009 [14]: The presence of any 3 of 5 risk factors constitutes a diagnosis of metabolic syndrome. 1. Elevated waist circumference (population- and country-specific definitions): ≥ 90 cm for men; ≥ 80 cm for women (Asians; Japanese; South and Central Americans); 2. Elevated triglycerides (drug treatment for elevated triglycerides is an alternate indicator): ≥ 150 mg/dL (1.7 mmol/L); 3. Reduced HDL-C (drug treatment for reduced HDL-C is an alternate indicator): < 40 mg/dL (1.0 mmol/L) in men; < 50 mg/dL (1.3 mmol/L) in women; 4. Elevated blood pressure (antihypertensive drug treatment in a patient with a history of hypertension is an alternate indicator): Systolic ≥ 130 and/or diastolic ≥ 85 mm Hg; 5. Elevated fasting glucose (drug treatment of elevated glucose is an alternate indicator): ≥ 100 mg/dL;

### Statistical analysis

Descriptive statistics were calculated for all the variables, including continuous variables (reported as mean values and standard deviations) and categorical variables (reported as numbers and percentages). Differences among categories were evaluated using t-test, ANOVA, non-parameter test or the χ^2^-test as appropriate. We used logistic regression analyses to estimate odds ratio (ORs) and 95% confidence intervals (CIs) for the possible risk factors of MetS after adjusting for possible confounders. All the statistical analyses were performed using SPSS version 17.0 software, and P values less than 0.05 were considered to be statistically significant.

## Results

### Changes of biochemical parameters from baseline to follow-up in different gender among residents with MetS in baseline

At baseline, 3167 residents were diagnosed MetS. We intend to estimate the changes of their metabolic parameter like SBP, BDP, WC and biochemical indexes in the past years. Data was shown in Table 1. At baseline, Male residents with Mets had higher value of SBP, DBP, BMI, WC, TG, FPG, UA and eGFR than female residents. On the contrary, female had relatively higher value of LDL-C, and HDL-C when compared with male. Similarly, in the follow-up, comparison of this parameters between female and male showed the same results as in the baseline except that there is no significant difference of eGFR between female and male in follow-up (*P *= 0.141). When compared data of baseline to follow-up, we can see there was apparent decrease of SBP, DBP and HDL in both gender. And discrepancies were observed in BMI, LDL-C and eGFR in different gender. BMI increased in male but decreased in female while others decreased in male and increased in female (LDL-C and eGFR). WC increased in follow-up compared with baseline in both genders. TG significantly increased while FPG significantly decreased in follow-up in comparison with baseline in female only.

**Table 1:** Metabolic parameters changes from 2012–2013 to 2015–2017 in MetS residents in baseline

	Male		Female		Total	
	Baseline	Follow-up	Baseline	Follow-up	Baseline	Follow-up
SBP (mmHg)	152.72 ± 21.44	147.67 ± 21.44*	149.86 ± 23.31	143.41 ± 23.06*	151.11 ± 22.71	145.14 ± 22.55*
DBP (mmHg)	89.16 ± 11.49	88.04 ± 12.28*	84.51 ± 11.20	83.35 ± 12.09*	86.26 ± 11.53	85.11 ± 12.37*
BMI (kg/m^2^)	27.24 ± 3.23	28.15 ± 3.74*	26.68 ± 3.47	25.78 ± 3.75*	26.89 ± 3.39	26.67 ± 3.92*
WC (cm)	91.58 ± 8.41	93.42 ± 8.59*	86.71 ± 8.31	89.44 ± 8.88*	88.54 ± 8.67	90.94 ± 8.98*
TG (mmol/L)	2.63 ± 2.32	2.59 ± 2.29	2.15 ± 1.61	2.28 ± 1.03^#^	2.63 ± 2.31	2.40 ± 2.02
LDL-C(mmol/L)	3.11 ± 0.88	3.06 ± 0.92^#^	3.22 ± 0.91	3.26 ± 0.93^#^	3.18 ± 0.90	3.18 ± 0.93
HDL-C (mmol/L)	1.20 ± 0.33	1.12 ± 0.34*	1.27 ± 0.29	1.21 ± 0.33*	1.25 ± 0.31	1.17 ± 0.34*
FPG (mmol/L)	6.61 ± 2.22	6.35 ± 2.18	6.43 ± 1.98	6.27 ± 2.08*	6.50 ± 2.07	6.35 ± 2.18*
UA (mmol/L)	359.99 ± 84.02	357.29 ± 82.60	273.52 ± 70.49	274.19 ± 66.15	305.94 ± 86.63	305.35 ± 83.13
eGFR (mi/min/1.73 m^2^)	92.35 ± 14.13	90.63 ± 14.61*	89.35 ± 16.47	91.45 ± 14.52*	90.47 ± 15.71	91.15 ± 14.56^#^
Current smoking (%)	52.4%	50.5%	16.0%	15.3%	29.6%	28.5%
Current drinking (%)	45.0%	45.3%	2.7%	3.6%^#^	18.5%	19.2%

*SBP* systolic blood pressure, *DBP* diastolic blood pressure, *TC* total cholesterol, *TG* triglyceride, *LDL-C* low-density lipoprotein cholesterol, *HDL-C* high-density lipoprotein cholesterol, *FPG* fasting plasma glucose, *UA* uUric acid

* Means P < 0.001 compared to baseline; # means P < 0.05 compared to baseline

### Dietary pattern changes from baseline to follow-up among residents with MetS in baseline

**Fig. 1:**
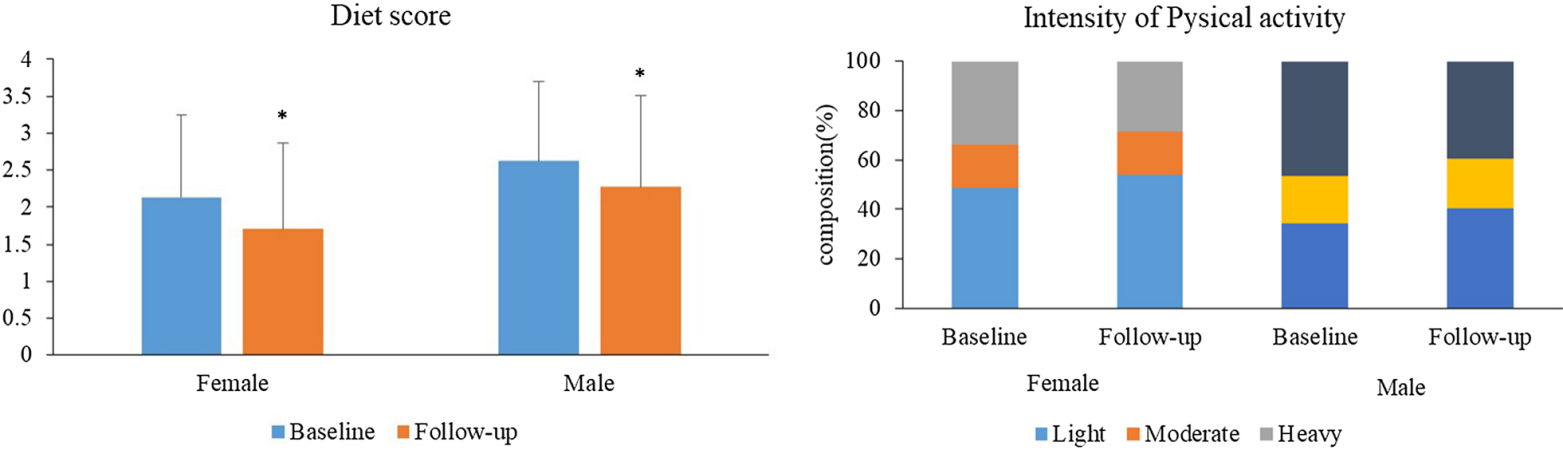
Gender difference of cumulative incidence of MetS during the 4.66 years median follow-up in different numbers of metabolic disorders in baseline

Figure 1 shown the changes of dietary pattern and intensity of physical activity from baseline to follow-up. The mean value of diet score in baseline was 2.13 ± 1.11 in female and 2.62 ± 1.08 in male. In both gender, the diet score significantly decreased in follow-up (1.70 ± 1.16 for female; 2.27 ± 1.24 for male). The higher value of diet score suggested a higher consumption of meat and lower consumption of vegetable. As for physical activity, we can see there is a significantly increase of light and decrease of heavy intensity of physical activity in the follow-up in both genders.

### Cumulative incidence of MetS among residents without Mets in baseline

**Fig. 2:**
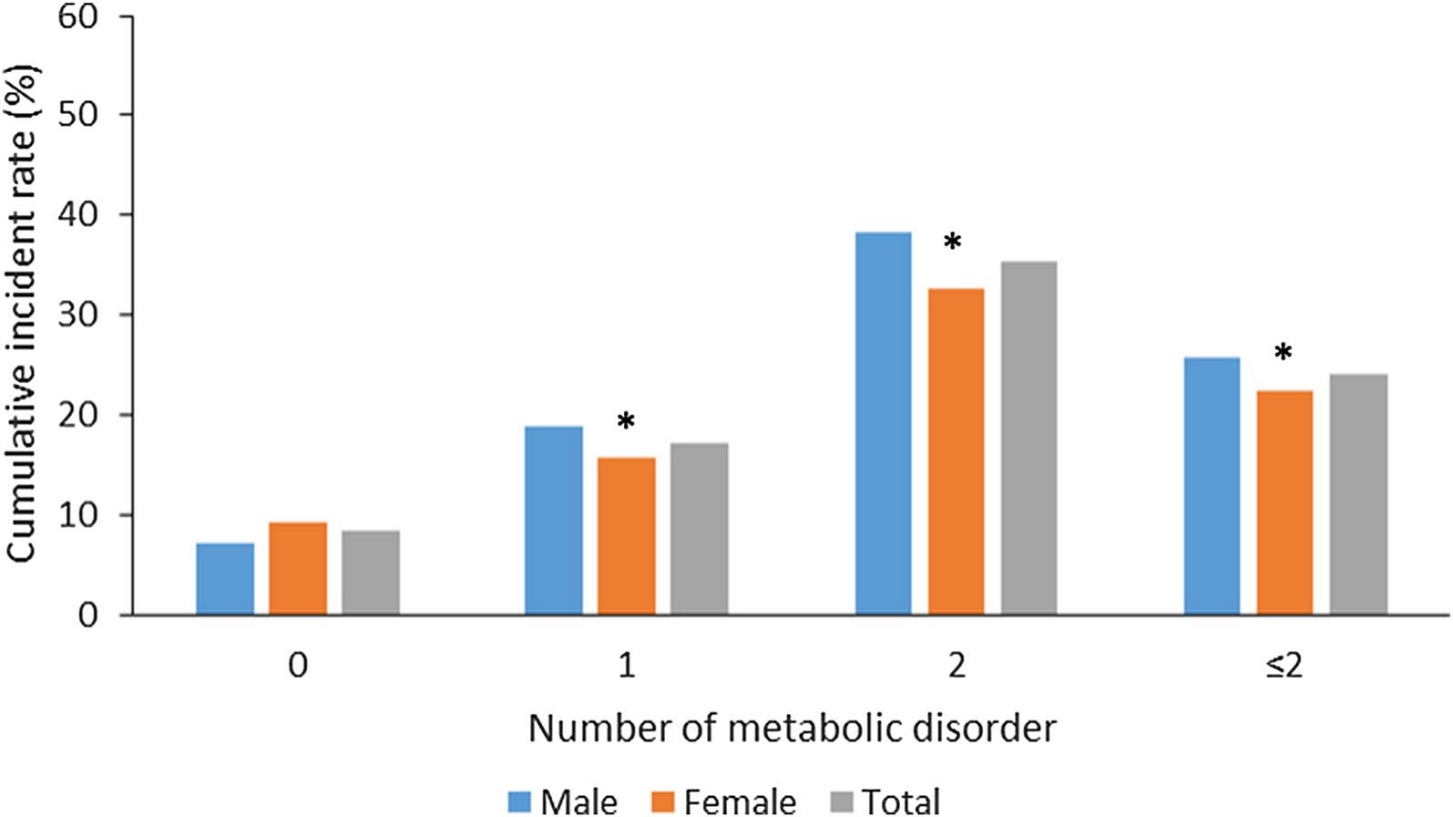
Changes of diet pattern and intensity of physical activity from baseline to follow-up

In present study, 4980 participants in baseline did not diagnosed of Mets. After the median 4.6 years follow-up, 1194 participants were newly diagnosed MetS (total: 24.0%; female: 22.3%; male: 25.8%). Besides, in Fig. 2. There is an increasing trend of MetS from participants without metabolic disorders to those with 2 metabolic disorders in baseline. Besides, female had significantly higher incidence of MetS than male in all the groups except for participant without metabolic disorder at baseline.

### Baseline characteristics of newly diagnosed MetS at follow-up

In Table 2. We can see newly diagnosed MetS were more likely to be women, older aged. MetS had higher rate of both primary school or below and high school or above compared with Non- MetS. Besides, MetS residents were less like to have severe physical activity. As for metabolic disorders, MetS had significantly higher baseline value of SBP, DBP, BMI, WC, TC, TG, LDL-C and FPG whereas significantly lower HDL-C. However, there is no difference existed among current smoking and drinking between MetS and Non-MetS.

**Table 2:** Baseline characteristics of rural Northeast Chinese residents by MetS status at follow-up overall and by non-MetS and MetS among residents without MetS at baseline

Variables	Total (n = 4980)	Non-MetS (n = 3786)	MetS (n = 1194)	P-value
Man	2586 (51.9)	2010 (53.1)	576 (48.2)	0.002
Age (years)	52.65 ± 10.21	52.20 ± 10.28	54.08 ± 9.89	< 0.001
Ethnicity				0.216
Han	316 (6.3)	234 (6.2)	82 (6.9)	
Others^a^	4664 (93.7)	3552 (93.8)	1112 (93.1)	
Education status				0.002
Primary school or below	2331 (46.8)	1720 (45.4)	611 (51.2)	
Middle school	2167 (43.5)	1689 (44.6)	478 (40.0)	
High school or above	482 (9.7)	377 (10.0)	105 (21.8)	
Physical activity				0.002
Light	1537 (31.2)	1123 (29.9)	414 (35.1)	
Moderate	918 (18.6)	721 (19.2)	197 (16.7)	
Severe	2479 (50.2)	1910 (50.9)	569 (48.2)	
Annual income (CNY/year)				0.055
≤ 5000	532(10.7)	399 (10.5)	133 (11.1)	
5000–20,000	2801(56.3)	2101 (55.5)	700 (58.7)	
> 20,000	1644(33.0)	1284 (33.9)	360 (30.2)	
Current smoking status (Yes)	1928(38.7)	1486 (39.2)	442 (37.0)	0.089
Current drinking status (No)	1278(25.7)	970 (25.6)	308 (25.8)	0.466
Marriage (Yes)	4922(98.8)	3747(99.0)	1175 (98.4)	0.081
Sleep duration (h/day)	7.27 ± 1.65	7.27 ± 1.64	7.29 ± 1.69	0.746
SBP (mmHg)	136.55 ± 21.85	134.58 ± 21.05	142.79 ± 23.16	< 0.001
DBP (mmHg)	79.73 ± 11.00	78.92 ± 10.69	82.33 ± 11.58	< 0.001
BMI (kg/m^2^)	23.59 ± 3.21	23.16 ± 3.15	24.93 ± 3.03	< 0.001
WC (cm)	78.39 ± 8.38	77.28 ± 8.06	81.94 ± 8.40	< 0.001
TC (mmol/L)	5.10 ± 0.99	5.04 ± 0.96	5.31 ± 1.05	< 0.001
TG (mmol/L)	1.14 ± 0.75	1.07 ± 0.55	1.36 ± 1.14	< 0.001
LDL-C (mmol/L)	2.83 ± 0.76	2.75 ± 0.73	3.06 ± 0.80	< 0.001
HDL-C (mmol/L)	1.52 ± 0.39	1.55 ± 0.39	1.46 ± 0.37	< 0.001
FPG (mmol/L)	5.50 ± 1.06	5.47 ± 1.03	5.60 ± 1.14	< 0.001

Data are expressed as the mean ± SD or as n (%)

*BMI* body mass index, *WC* waist circumference, *CNY* China Yuan (1CNY = 0.161 USD), *SBP* systolic blood pressure, *DBP* diastolic blood pressure, *TG* triglyceride, *LDL-C* low-density lipoprotein cholesterol, *HDL-C* high-density lipoprotein cholesterol, *FPG* fasting plasma glucose

^a^Including some ethnic minorities in China, such as Mongol and Manchu

### Cumulative incidence rate of MetS by different status markers and the possible risk factors associated with higher incidence of MetS

Table 3 showed that there is discrepancy of incidence of MetS among female and male. In female, there is a significantly increasing incidence of MetS as the age increased. Female residents who have more than 1 children also had significantly high incidence of MetS compared to those with one or less child. With the education level increased from primary school or below to high school or above, the incidence of MetS also decreased in female. In male, residents without marriage have relatively higher incidence of MetS than those married. Besides, there is contrary result existed in current smoking status, compared to non-current smoker, male current smoker has low rate of MetS while female current smoker had higher incidence rate. As for drinking status, current drinker had significantly higher incidence of MetS than non-current drinkers in male. In male, the single associated risk factors of higher incidence of MetS was current drinking while married status and current smoking were associated with lower incidence. However, in female, increasing age, ≥ 4 times/w bean and bean product consumption and tea intake were related with higher incidence of MetS.

**Table 3:** Cumulative incidence rate of MetS by levels of different status markers and multiple logistic regression analysis of MetS incidence and the associated factors in the rural population of Liaoning Province, China

	Male			Female			Total		
	N	Cumulative incidence (%)	OR (95% CI)	N	Cumulative incidence (%)	OR (95% CI)	N	Cumulative incidence (%)	OR (95% CI)
Age									
35–44	124	21.4	1.00 (reference)	*124*	*16.2*	1.00 (reference)	*248*	*18.5*	1.00 (reference)
45–54	166	21.3	1.02 (0.78,1.43)	*222*	*26.5*	1.78 (1.37,2.31)	*388*	*24.0*	*1.40 (1.16,1.69)*
55–64	197	23.9	1.13 (0.85,1.50)	*199*	*33.6*	2.41 (1.78,3.25)	*396*	*28.0*	*1.69 (1.37,2.07)*
≥ 65	89	22.1	1.02 (0.70,1.48)	*73*	*36.7*	2.61 (1.73,3.95)	*162*	*26.9*	*1.57 (1.19,2.07)*
Number of child									
≤ 1	235	21.8	1.00 (reference)	*257*	*23.4*	1.00 (reference)	*492*	*22.6*	1.00 (reference)
> 1	341	22.6	1.04 (0.85,1.29)	*361*	*27.8*	0.97 (0.79,1.19)	*702*	*25.0*	1.00 (0.87,1.16)
Ethnicity									
Hank	538	22.2	1.00 (reference)	574	25.6	1.00 (reference)	1112	23.8	1.00 (reference)
Others^a^	38	23.6	1.04 (0.71,1.54)	44	28.4	1.11 (0.76,1.62)	82	25.9	1.19 (0.84,1.44)
Education status									
Primary school or below	245	22.5	1.00 (reference)	*366*	*29.5*	1.00 (reference)	*611*	*26.2*	1.00 (reference)
Middle school	273	22.1	0.99 (0.80,1.22)	*205*	*22.0*	0.91 (0.73,1.13)	*478*	*22.1*	0.93 (0.80,1.08)
High school or above	58	22.4	1.04 (0.73,1.47)	*47*	*21.1*	0.82 (0.56,1.19)	*105*	*21.8*	0.89 (0.69,1.14)
Physical activity									
Light	150	22.8	1.00 (reference)	*264*	*30.0*	1.00 (reference)	*414*	*26.9*	1.00 (reference)
Moderate	90	19.7	0.88 (0.64,1.19)	*107*	*23.3*	0.79 (0.60,1.04)	*197*	*21.5*	*0.81 (0.66,0.99)*
Severe	327	22.6	1.06 (0.82,1.35)	*242*	*23.4*	0.83 (0.67,1.04)	*569*	*23.0*	0.92 (0.78,1.08)
Annual income (CNY/year)									
≤ 5000	72	22.6	1.00 (reference)	61	28.6	1.00 (reference)	133	25.0	1.00 (reference)
5000–20,000	336	23.0	1.06 (0.78,1.43)	364	27.1	1.10 (0.78,1.55)	700	25.0	1.08 (0.86,1.35)
> 20,000	168	20.8	0.94 (0.67,1.32)	192	22.9	1.01 (0.70,1.46)	360	21.9	0.97 (0.76,1.24)
Sleep duration (h/day)									
≤ 7	260	21.8	1.00 (reference)	316	25.0	1.00 (reference)	576	23.5	1.00 (reference)
7–8	171	22.0	1.02 (0.81,1.26)	190	27.6	1.34 (1.07,1.67)	361	24.6	1.14 (0.98,1.33)
8–9	91	22.9	1.05 (0.79,1.39)	70	25.1	1.08 (0.79,1.47)	161	23.8	1.06 (0.86,1.30)
> 9	53	24.5	1.22 (0.86,1.73)	41	25.6	1.16 (0.78,1.72)	94	25.0	1.16 (0.89,1.50)
Marriage									
No	*19*	*37.3*	1.00(reference)	0	0	1.00 (reference)	19	32.8	1.00(reference)
Yes^b^	*557*	*22.0*	*0.46(0.25,0.85)*	618	25.9	-	1175	23.9	0.57(0.32,1.03)
Current smoking status									
No	*252*	*24.0*	1.00 (reference)	*500*	*25.0*	1.00 (reference)	752	24.6	1.00 (reference)
Yes	*324*	*21.1*	*0.81 (0.67,0.99)*	*118*	*30.1*	1.09 (0.84,1.41)	442	22.9	0.94 (0.81,1.11)
Current drinking status									
No	*287*	*20.8*	1.00 (reference)	599	25.8	1.00 (reference)	886	23.9	1.00 (reference)
Yes	*289*	*23.9*	*1.23 (1.01,1.50)*	19	26.8	0.80 (0.46,1.42)	308	24.1	1.17 (0.98,1.41)
Beans or Bean product									
Rarely	173	20.3	1.00 (reference)	254	25.1	1.00 (reference)	427	22.9	1.00 (reference)
2–3 times	324	23.3	1.18 (0.95,1.46)	286	25.2	1.02 (0.84,1.25)	610	24.1	1.10 (0.95,1.28)
≥ 4 times	75	22.7	1.15 (0.84,1.58)	76	31.7	*1.43 (1.04,1.96)*	151	26.5	*1.26 (1.01,1.57)*
Tea intake (Frequency/day)									
No	173	20.3	1.00 (reference)	422	24.4	1.00 (reference)	685	22.8	1.00 (reference)
Rarely	324	23.3	1.22 (0.96,1.53)	115	28.5	*1.37 (1.07,1.77)*	269	26.0	*1.29 (1.09,1.53)*
1–2 times	75	22.7	1.12 (0.87,1.43)	75	31.6	*1.43 (1.05,1.95)*	208	25.8	*1.24 (1.02,1.50)*
3–4 times	572	22.2	1.09 (0.67,1.77)	6	26.1	1.29 (0.49,3.38)	31	23.3	1.09 (0.71,1.68)

Adjusted for Cardiovascular diseases (angina, myocardial infarction, arrhythmia, and heart failure), cerebrovascular diseases (cerebral hemorrhage, cerebral infarction, subarachnoid hemorrhage, Transient Ischemic Attack) and chronic kidney diseases (nephritis, acute/chronic renal failure)

*OR* odds ratio, *95% CI* 95% confidence interval

Italics means P < 0.05; CNY, China Yuan (1CNY = 0.161 USD)

^a^Including some ethnic minorities in China, such as Mongol and Manchu

^b^Including widowed, divorced/separated and unmarried

## Discussion

In the present study, we reported the changes of metabolic parameters. In male with MetS at baseline, BMI and WC significantly increased in the follow-up whereas HDL, blood pressure and LDL decreased. While in female with MetS at baseline, WC, TG and LDL increased but HDL, blood pressure, FPG and BMI all decreased in follow-up. Diet score significantly lower in both gender at follow-up while residents had higher light intensity than they had in baseline. The cumulative incidence of MetS was higher in female than in male. Besides, with the increase metabolic disorders in baseline, the incidence of MetS also increased. Gender discrepancy existed among the risk factors of developing MetS. In male, current drinking was risk factors while current smoking and married status were protective factors. In female, bean and bean product consumption and tea consumption were associated with higher risk of developing MetS.

NHNES data found that average BMI increased by 0.37% per year in both male and female while WC increase 0.37 and 0.27% per year in female [7]. While in our study, among previous diagnosed MetS residents, WC increased during follow-up in both genders whereas BMI increased in male but decreased in female. This is might be due to the increasing age. Previous studies concluded that rate of abdominal obesity increased with age [15, 16]. Also it might be relevant to the decrease rate high and moderate physical activity intensity. MetS is associated with physical inactivity and unhealthy diet [19]. From data of our study, diet score decreased significantly during follow-up. This might be helpful to control hypertension, dyslipidemia and diabetes. Blood pressure, both systolic and diastolic blood pressure significantly decreased in the follow-up in both gender. As diet pattern index, diet score inferred the composition of diet. The higher score means more meat and less vegetable which are more similar to Western diet while lower score are more likely Mediterranean diet. Courtney R Davis and colleagues confirmed that Mediterranean diet help to lower blood pressure and improved endothelial function [17]. Besides, more vegetable and less meat diet showed great improvement of high glucose level and HbA1c level [18]. Adherence to a healthy diet has been proved to be associated with lower risk of MetS in both developing and developed countries [19, 20]. On interesting thing in the follow-up is that dyslipidemia was even worse in female residents with previously diagnosed MetS. The value of TG and LDL-C increased while HDL-C decrease significantly in follow-up. This might be due to the larger rate of postmenopausal female in the follow-up. A cumulative evidence inferred that menopause status had close relationship with dyslipidemia in female [21]. Increased prevalence of dyslipidemia was associated not only with the post-menopausal stage but also late menopausal transition period [22]. In the present study, 177 female residents transited to post-menopause during the past years and 1544 in total were post-menopause. Therefore, unlike in male, lipid parameters increased in female.

Hongge Sun and colleagues reported an 18.55% cumulative incidence of MetS during the 7 years of follow-up in Chinese residents [23]. Adriano M Pimenta also claimed 6.0% cumulative incidence of MetS during the 8.3 years of follow-up in Spanish [24]. As for our present study, the cumulative incidence of MetS was 24.0% during the 4.3 year of follow-up which was significantly higher than many previous studies. However, Yazd Health Heart Project reported a relatively higher incidence with 56.1% after 10 year follow-up and concluded that increased risk of MetS was in those did not usually eat salad and did not try to control their body [25]. While in our study, there is discrepancy of risk factors among female and male residents. In male residents without MetS at baseline, the only risk factor that has statistical difference is current drinking status. One recent review enrolled six prospective studies, 28,862 participants with 3305 cases of MetS, concluded that heavy alcohol consumption was associated with an increased risk of MetS while very light alcohol consumption decreased the risk of Mets [26]. However, in our present study, we are lack of the dose of alcohol that residents took. One interesting finding is that in our study, baseline current smoking was related with lower incidence of MetS at follow-up. It is contrary to many previous studies. We retrospect analyzed the male residents that smoking in baseline, found that they were younger than non-current smokers (53.27 ± 10.02 vs. 55.70 ± 11.12, *P *< 0.001). Current smoker had relatively lower value of SBP, DBP, WC compared to non-current smokers. Besides, at baseline, current smoker had significantly less number of metabolic disorder than non-current smokers (1.25 ± 0.73 vs. 1.36 ± 0.69, P < 0.001). In all, they might be reasonable that in comparison with current smokers in baseline, non-current smokers had higher risk of MetS in fellow-up. Married male residents in the present seemed have lower incidence of MetS compared with non-married ones. This finding was consistence with the survey among Korean middle-aged women that prevalence of MetS was 30% in the married group and 34.2% in the unmarried group [27].And the possible explanation might be related to the low socioeconomic status among unmarried male in the present study. Those unmarried male were likely to be low income family (51.0% vs. 11.9%, *P *< 0.001) and higher rate of low educational level (70.6% vs. 41.6% for primary school and under) compared with married male. Accumulative evidence inferred that a low socioeconomic status increased the risk of cardiovascular diseases [28, 29]. In male, the major associated factors of Mets were unhealthy living habit while in female are more likely to be the diet habit. In female, increasing age is a significant risk factors for MetS. Female aged over 65 years had 2.61-fold of risk to have MetS. Besides, consumption of bean and bean product and tea also associated with higher incidence of MetS. This findings are contrary to many previous study held in both human and animal claiming a beneficial effect of tea on metabolic disorders [30–32]. But the specific reasons to explain this inconsistent results in our study are still unclear. Maybe further studies are required to figure out the possible reasons.

The present study has many limitation. First, the incidence of MetS was based on a single blood test which might have bias. Second, we only evaluate whether residents are current smoker or drinker. We did not estimate the exact dose of the smoking and drinking. Hence, there might be bias to conclude that drinking or smoking are risk or beneficial factors of MetS.

## Conclusion

In conclusion, the present study estimates the changes of metabolic parameters of residents with MetS at baseline and with the conclusion that many metabolic index did alleviated in follow-up especially the blood pressure. Besides, among residents without MetS at baseline, the cumulative incidence of MetS still high in rural areas of China. The gender discrepancy in the risk factors of MetS help us better knowing what are the major aspects that we should focus on and what can be modulated in order to decrease MetS in rural China.

## Data Availability

Enquiries regarding the availability of primary data should be directed to the principal investigator Professor Yingxian Sun (sunyingxiancmu1h@163.com).

## Abbreviations

MetS: Metabolic syndrome
OR: Odds ratio
BMI: Body mass index
SBP: Systolic blood pressure
DBP: Diastolic blood pressure
FPG: Fasting plasma glucose
TC: Total cholesterol
LDL-C: Low-density lipoprotein cholesterol
HDL-C: High-density lipoprotein cholesterol
TG: Triglyceride
eGFR: Estimated glomerular filtration rate
